# Validation of the Acoustic Breathiness Index in Speakers of Finnish Language

**DOI:** 10.3390/jcm12247607

**Published:** 2023-12-10

**Authors:** Elina Kankare, Anne-Maria Laukkanen

**Affiliations:** 1 Department of Rehabilitation and Psychosocial Support, Logopedics, Phoniatrics, Tampere University Hospital, 33520 Tampere, Finland; 2 Speech and Voice Research Laboratory, Tampere University, 33100 Tampere, Finland; anne-maria.laukkanen@tuni.fi

**Keywords:** dysphonia, hoarseness, GRBAS, acoustic noise detection

## Abstract

Breathiness (perception of turbulence noise in the voice) is one of the major components of hoarseness in dysphonic voices. This study aims to validate a multiparameter analysis tool, the Acoustic Breathiness Index (ABI), for quantification of breathiness in the speaking voice, including both sustained vowels and continuous speech. One hundred and eight speakers with dysphonia (28 M, 80 F, mean age 50, SD 15.4 years) and 87 non-dysphonic controls (18 M, 69 F, mean age 42, SD 14 years) volunteered as participants. They read a standard text and sustained vowel /a:/. Acoustic recordings were made using a head-mounted microphone. Acoustic samples were evaluated perceptually by nine voice experts of different backgrounds (speech therapists, vocologists and laryngologists). Breathiness (B) from the GRBAS scale was rated. Headphones were used in the perceptual analysis. The dysphonic and non-dysphonic speakers differed significantly from each other in the auditory perceptual evaluation of breathiness. A significant difference was also found for ABI, which had a mean value of 2.26 (SD 1.15) for non-dysphonic and 3.07 (SD 1.75) for dysphonic speakers. ABI correlated strongly with B (r_s_ = 0.823, *p* = 0.01). ABI’s power to distinguish the groups was high (88.6%). The highest sensitivity and specificity of ABI (80%) was obtained at threshold value 2.68. ABI is a valid tool for differentiating breathiness in non-dysphonic and dysphonic speakers of Finnish.

## 1. Introduction

### 1.1. What Is Breathiness

Breathiness is a characteristic in many disordered voices [1,2]. It occurs in organic voice disorders as well as in functional or neurological voice disorders [1,3]. Some breathiness can also be heard in the softly produced healthy voice, especially in women [4,5]. Breathiness refers to the auditory perception of air turbulence and is caused by air leakage from the glottis [6,7,8]. 

### 1.2. Perceptual Tools to Detect Breathiness

Many auditory perceptual tools have been developed to evaluate voice quality and at the same time breathiness in the voice. These tools include, for example, the GRBAS scale [6], the Australian Perceptual Voice Profile [9], the Swedish Stockholm Voice Evaluation [10], the CAPE-V (Consensus Auditory Perceptual Evaluation of Voice) [8] and the Danish Dysphonia Assessment [11]. Although an experienced listener can estimate the amount of breathiness in the voice by perceptual analysis, this is a subjective estimation [12]. An objective measure to evaluate voice quality and the amount of breathiness is needed, especially in clinical work and in the rehabilitation of patients with varying voice disorders [13,14]. 

### 1.3. Acoustic Tools to Detect Breathiness

Various signal analysis methods have been applied to predict perceived breathiness from acoustic characteristics. All of the methods aim to measure the amount of periodic and non-periodic components in a sound signal. These methods include, e.g., harmonic-to-noise ratio (HNR) [15], noise-to-harmonic ratio (NHR), voice turbulence index and soft phonation index [16,17], signal periodicity, first harmonic amplitude and spectral tilt and cepstral peak prominence (CPP and its smoothed version CPPS) [1,18]. Periodicity or, rather, reduced periodicity with increased jitter and shimmer (i.e., irregular variation in period duration or amplitude, respectively) has been found to predict well perceived breathiness both in non-dysphonic and in dysphonic voices [1,18]. On the other hand, jitter and shimmer are characteristics that are related to irregular vocal fold vibration, whose main perceptual correlate is “roughness” [6]. Furthermore, jitter, shimmer and spectrum based measures of noise, like HNR, are affected by the pitch and intensity of the voice, which impairs their reliability in dysphonia detection [19,20,21]. CPPS, which is based on the spectrum of the logarithmic spectrum [18], is independent of pitch and intensity. It has been found to show highest correlations with perceived hoarseness, roughness and breathiness [22,23]. Thus, there seemed to be a need to develop an index that would be able to focus more on the acoustic characteristics of breathiness rather than those of roughness which refers to irregular vocal fold vibration, and to distinguish non-dysphonic and dysphonic breathiness. The Acoustic Breathiness Index (ABI) has been developed to meet these needs [12,24]. 

### 1.4. What Is ABI?

The ABI is a multidimensional method with nine separate acoustic measures for detecting breathiness in the voice. Measures used in the ABI are smoothed cepstral peak prominence (CPPs), jitter local (Jit), glottal-to-noise excitation ratio (GNE), high frequency noise of 6000 Hz (Hfno), harmonic-to-noise ratio of Dejonckere (HNR-D), the amplitude difference between the first two harmonics (H1-H2), two measures of shimmer (Shim dB and Shim%) and period standard deviation (PSD) [12]. CPPs is the distance between the cepstral peak that corresponds to the first harmonic and the point with equal quefrency (inverse of frequency) on the regression line through the smoothed cepstrum [12]. The higher the value of CPPs, the more periodic, i.e., the clearer and more noiseless, the sound is in terms of auditory perception. CPPs is affected by both turbulence noise and signal perturbation (jitter and shimmer). Jit, i.e., jitter local, is the mean difference between successive periods, divided by the average period length. GNE [25] indicates whether a voice signal originates from vocal fold vibrations or from turbulent noise. GNE is independent of jitter and shimmer. A clear, nonbreathy voice results in high GNE. Hfno (up to 6000 Hz) indicates the spectral level difference between the ranges of 0–6 kHz and 6–10 kHz. A breathy voice with more noise in the high-frequency range has a smaller Hfno. HNR-D from Dejonckere and Lebacq [26] analyses the harmonic structure against noise in the long-term average spectrum in the formant zone between 500 Hz and 1500 Hz. A cepstrum is calculated to determine F0. A higher value of HNR-D indicates a less breathy voice. H1–H2, i.e., the difference in level between the first two harmonics, is greater in breathy voices. Shimmer measures the amplitude perturbation through the difference between successive periods divided by the mean amplitude. The value is calculated both in dB and in percentages. PSD is a perturbation measure revealing the variation in the standard deviation of periods [12].

Since the ABI is calculated from both sustained vowels and continuous speech, it needs to be validated in different languages [24]. The ABI has so far been validated in eight different languages, Dutch [12], German [24], Japanese [27], Korean [28], Brazilian Portuguese [29], Spanish [30], South Indian [31] and Persian [32].

### 1.5. Aim and Research Questions of the Present Study

The present article introduces a study where we aimed to validate the ABI in a Finnish speaking population. In this study, we sought an answer to two main questions: (1) Is the ABI a valid robust method to distinguish dysphonic breathy voice quality in a Finnish speaking population? (2) What is the best threshold value for ABI analysis in a Finnish speaking population?

## 2. Materials and Methods

### 2.1. Participants

The present study applied the ABI and auditory perceptual evaluation of breathiness to 195 Finnish speaking participants. The voice material of this study is the same as in the validation study of the Acoustic Voice Quality Index version 03.01 in Finnish. 

### 2.2. Dysphonic Participants

One hundred and eight dysphonic participants were volunteer patients in the phoniatric department in the university hospital. Twenty-eight of the patients were males (mean age 51 years, SD 13.8, range 19–75). Eighty of the patients were females (mean age 51 years, SD 16.2, range 19–84). Table 1 shows the diagnoses of the participants. 

### 2.3. Non-Dysphonic Controls

Eighty-seven vocally healthy persons with no diagnosis of dysphonia participated as controls. Eighteen of the participants were males (mean age 49 years, SD 9.9, range 32–60), and 69 were females (mean age 40 years, SD 14.5, range 19–67). Seventy-nine of the healthy participants scored under 38 points on the VAPP questionnaire (Voice Activity and Participation Profile) [33] and eight of them scored over 38 which has been considered the threshold value for voice disorder [34]. However, all participants considered themselves to be vocally healthy.

### 2.4. Recordings

All voice samples were recorded with an AKG C544L (AKG, Vienna, Austria) head-mounted condenser microphone with the Focusrite iTrack Solo (Focusrite PLC, High Wycombe, England) audio interface and using Praat software (version 6.2.23) in the computer. The recording used a sampling frequency of 44.1 kHz and the amplitude resolution was 16 bits. The distance of the microphone was 4 cm from the right side of the corner of the mouth at an angle of 45 degrees. The distance and position of the microphone were checked for each participant by measuring the distance from the corner of the mouth with a ruler. 

The voice material for the study was collected for patients and 49 vocally healthy participants in a quiet treatment room at Tampere University Hospital. Thirty-eight of the healthy voices were recorded in studio conditions at Tampere University. The mean signal-to-noise ratio of the recordings (i.e., the difference in level between the sample and that of the background noise level) was 39.8 dB (SD 5.6 dB). In all samples the SNR was well over the recommended norm of SNR > 30 dB, so it can be confirmed that the recording conditions were acceptable.

### 2.5. Voice Samples

As voice samples, the standard text “Pohjantuuli ja aurinko” (North wind and the sun) was read aloud and a sustained vowels [a:] was produced three times. The participants were asked to use a voice pitch and intensity that suited them best, and the length of the sustained vowel was suggested to be five seconds. The participants were asked to produce the vowel in a spoken manner rather than singing.

For the ABI analysis the first 31 syllables from the read text and three seconds from the middle of the second sustained vowel were used. In the Finnish AVQI validation study, it was confirmed that 31 syllables of Finnish language text readings correspond on average to three-second long vowels [35]. The confirmation of the 31 syllables was executed for Finnish language the same way as described when finding out the syllable count of the Dutch sample [36]. Therefore, the index to be obtained would consist of a balanced duration of speech and sustained vowel phonation. For the analysis, connected speech sample was marked “cs” and the three-second sustained vowel sample “sv”. The ABI analysis was executed with VOXplot Acoustic Voice Quality Analysis software, version 2.0.0 [12,37]. The equation to calculate the ABI in the VOXplot software was the one presented by Barsties v. Latoszek et al. 2017 [12]: ABI = (5.0447740915 − [0.172 × CPPs] − [0.193 × Jit] − [1.283 × GNEmax − 4500 Hz] − [0.396 × Hno − 6000 Hz] + [0.01 × HNR –D] + [0.017 × H1 − H2] + [1.473 × Shim − dB] − [0.088 × Shim] − [68.295 × PSD]) × 2.9257400394. The ABI analysis gives the result of an index value between 0 and 10, a value of 0 meaning that there is no breathy sound in the voice and the higher the index number, the breathier sound there is in the voice sample.

### 2.6. Auditory–Perceptual Analysis

In order to validate the ABI analysis of the Finnish language, a listening analysis was performed. Nine voice experts in the field of voice (three phoniatrician/otolaryngologists, three speech therapists and three vocologists) listened to the voice samples and gave their evaluation of B from the GRBAS scale [6]. The scale is from 0 to 3, 0 signifying “no breathiness at all” and 3 signifying “very much breathiness” in the voice. The listening samples consisted of 31 syllables of continuous speech from the beginning of the text reading and three seconds of a sustained vowel. The length of one sample was thus six seconds and there was a total of 220 samples. For the intra-rater reliability analysis, 25 samples were rated twice. Before the listening test, listeners’ ears were calibrated with the anchor voice samples [38,39]. In the calibration, there were two anchor voice samples for each category of the degree of breathiness 0–3, i.e., there were in total eight anchor samples. The anchor voice samples were selected from the voice material of the present study by one experienced speech therapist and one experienced vocologist. The listening test was conducted on each listener’s own computer with around-ear headphones. Listeners received the voice material and instructions for the listening analysis on a memory stick. They were asked to make a judgement from a combination of continuous speech and sustained vowel phonation and mark the results in an Excel table. During the listening analysis the raters were able to listen to the samples as many times as they felt necessary; moreover, they were asked to listen to the anchor voice samples at least once after every 32 samples. This was carried out to prevent the listeners from losing focus and straining their hearing too much. Reminders about the anchors were marked in the Excel table as was the instruction to take a short break after listening to 128 samples, about halfway through the task. The interval for listening to the anchors was chosen on the basis of previous listening analyses. The listening analysis took on average from two to three hours. 

### 2.7. Statistical Analysis

The statistical analysis was conducted using SPSS for Windows version 26 (IBM Corp., Armonk, NY, USA). All the results were considered statistically significant at *p* ≤ 0.05. In the Finnish validation of the ABI, first the intra-rater reliability of the perceptual raters was analysed with the Cohen kappa (*C*κ) and secondly the raters’ inter-rater reliability was analysed with the Fleiss kappa (*Fκ*) [40]. Raters with intra-rater reliability *Cκ* ≥ 0.41 were selected for inclusion in the study. The inter-rater reliability between the perceptual rates was defined to be at least ≥0.41 [40]. Next in the validation process, the relationship between the mean values of ABI and the mean values of the perceptual evaluation of breathiness were tested with the Spearman’s rank order correlation coefficient (r_s_, r^2^). Finally, the diagnostic accuracy of the ABI was evaluated with ROC (receiver operating characteristic) curve. The diagnostic accuracy was evaluated according to the sensitivity of the ABI to distinguish between disordered voice and heathy voice, and specificity to detect voices without breathiness. A nonbreathy voice was defined as a voice that received a perceptual mean rating of B 0–0.49. Additionally, the ability of the ABI to distinguish between normal and dysphonic breathiness was evaluated by the area under ROC curve (A_ROC_). The ROC curve and the Youden index were used to differentiate the best threshold level for the ABI to differentiate healthy and dysphonic voices in the Finnish language. Likelihood ratios (LR+ and LR−) were used to differentiate the probability of persons with breathy voice having ABI value above the threshold level (LR+) or persons with nonbreathy voice having ABI value below the threshold level. To define the optimal threshold level, both the positive and negative likelihood ratio and the sensitivity/(1 − specificity) and (1 − sensitivity/specificity were used). 

## 3. Results

### 3.1. Reliability of the Perceptual Evaluation

**Figure 1:**
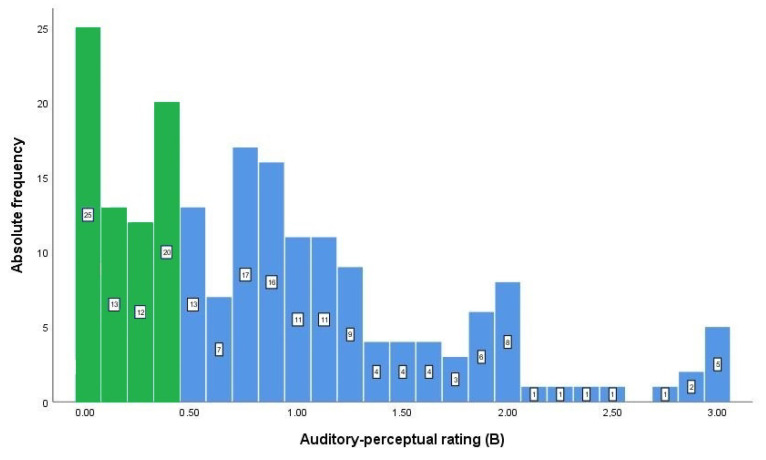
Frequency distribution of the mean breathiness by eight raters. Green colour on the graph indicates those participants who did not have breathiness (mean 0–0.49) in their voices and blue indicates those who were rated to have breathy voice quality (mean 0.50–3).

The intra-rater reliability of the listening analysis of the breathiness in C*k* was between 0.395 and 0.809. One rater, however, presented a C*k* value lower than the acceptable 0.41 and was excluded from the analysis. The remaining eight raters reported C*k* values between 0.451 and 0.809. This group of eight raters showed reasonable inter-rater reliability (*Fk* = 0.435) and therefore the mean of their listening analysis represents the auditory assessment of breathiness in this study. The mean distribution of the auditory perceptual rating is seen in Figure 1. It is possible to deduce from Figure 1 that some breathiness was also heard in some of the non-dysphonic voices.

The listeners in this study represented three different occupational groups: phoniatrician/otolaryngologists, speech therapists and vocologists. The vocologists’ evaluation differed significantly from that of the other two groups (Mann–Whitney U test, phoniatrician/otolaryngologists vs. vocologists *p* = 0.002, speech therapists vs. vocologists *p* = 0.000). Vocologists rated more breathiness in the voice than the raters in the other groups.

### 3.2. Results for the ABI and Perceptual Evaluation of Breathiness

**Figure 2:**
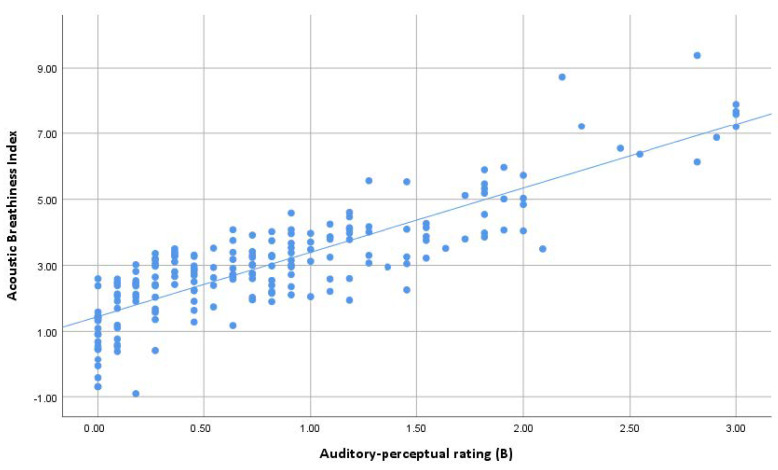
Scatter plot and the linear regression line between auditory–perceptual rating and ABI results.

ABI results correlated strongly with auditory perceptual rating of breathy voice quality (Spearman’s rho 0.823, *p* = 0.01) (Figure 2). Non-dysphonic and dysphonic groups differed significantly from each other in both ABI and perceptual results (Mann–Whitney test, *p* < 0.001) (Table 2). 

### 3.3. Sensitivity and Specificity of ABI

**Figure 3:**
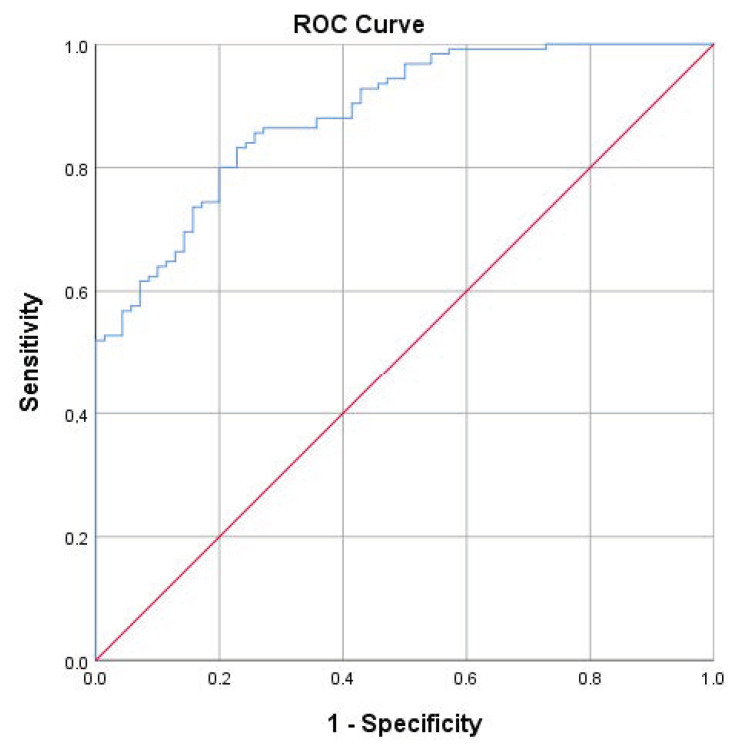
ROC curve analysis illustrating the diagnostic accuracy of ABI, area under ROC curve = 88.6% (A_ROC_ line blue, reference line red).

The ability of the ABI to distinguish between breathy and nonbreathy voices was evaluated with ROC analysis. A_ROC_ = 0.886 (i.e., 88.6%) showed high discriminatory power to distinguish nonbreathy voices from breathy voices (Figure 3). The highest Youden’s index was 0.60, where the best sensitivity of 80% and specificity of 80% were obtained at the cut point value 2.68. In the likelihood ratio the statistical guideline values were not reached (likelihood ratios LR+ 4.00 and LR− 0.25). Table 3 shows the threshold values of the eight previous ABI validation studies and the cut-point value of the Finnish validation study, as well as the statistical values of sensitivity, specificity, likelihood ratios, and the correlations between the ABI and the perceptual evaluation. 

## 4. Discussion

This study aimed to investigate whether the acoustic breathiness index (ABI) is a valid and robust method to distinguish dysphonic breathiness from healthy voices in a Finnish speaking population, and, if so, what the best threshold value for ABI would be. These research questions are important; while breathiness is one of the main characteristics in dysphonia and the first component of hoarseness [41], it is also frequently found in the non-dysphonic population. This requires a more focused, multi-parameter tool for the detection of true breathiness and to be able to distinguish dysphonic breathiness. Breathiness appears to be perceived better in females’ voices but reduced loudness of voice increases its presence in both genders [42]. It has been found to be related to perceptions of femininity and attractiveness in female voice quality [5,43], and it may also be related to attractiveness in male voices [44], although voice characteristics evoke different evaluations in different cultures [45,46]. 

Some breathiness was perceived in some of the healthy voices in the present study. This is to be expected, as breathiness is also a cultural characteristic. In particular, vocologists who work with normal and supranormal (trained) voices were more sensitive than clinicians (phoniatrician/otolaryngologists and speech therapists) in rating breathiness. The main reason for including raters from different professional groups was to get a larger distribution of evaluations, which would also take into account the existence of some breathiness in normal voices. Furthermore, breathiness was the only characteristic that was rated in the present study; thus, the raters had to focus on this particular characteristic, which the acoustic tool was also specifically developed to measure. 

The results of the present study show that the dysphonic voices scored significantly higher both on perceived breathiness and ABI, although the dysphonic group included patients with very different diagnoses and thus with different acoustic characteristics. This suggests that ABI measures what it is intended to measure. Perceived breathiness correlated strongly with ABI (rs 0.823, *p* 0.01) suggesting the ecological validity of the index. The discriminatory power of the ABI was high (88.6), showing that the method successfully differentiated between the dysphonic and non-dysphonic groups. The highest sensitivity (80%) and specificity (80%) in differentiation was obtained at ABI = 2.68. This can be thus used as a threshold for the clinical analysis of breathiness in a Finnish speaking population. Other studies [12,24,27,28,29,30,31,32] have reported slightly higher threshold values than what was found in the present study (Table 3). The reason may be related to language and cultural differences [29]. The Finnish language has a high prevalence of vowels, and a lack of linguistically breathy vowels that occur for instance in Gujarati, Mon-Khmer and Jalapa Mazatec [47], or sonorous fricatives. Finns may be more sensitive in perceiving breathiness. It may be speculated whether there could be a connection to some earlier findings where breathy voice quality seemed to convey an impression of emotional instability and implausibility of the speaker among Finnish listeners [46,48]. 

The mean age in the dysphonic group of the present study was somewhat higher than that of the non-dysphonic group and, in both groups, females were in the majority. These characteristics reflect the clinical reality that dysphonic patients are typically not very young and that females form the majority of voice patients [49,50,51]. However, earlier findings have shown that the ABI is not significantly dependent on age or sex [22,27]. 

The use of only one perceptual variable in the listening evaluation may be seen as a limitation in the study since then the presence of other potential characteristics of hoarseness remain unknown. This is, however, the policy that other ABI validation studies have adopted [12,24,28,30,32]. Further study of the average ABI results for different diagnostic groups is warranted. This would require larger numbers of participants in different diagnostic groups. Such a study should also address further the capability of listeners and ABI to differentiate between breathiness and other components of dysphonia by including other perceptual variables than merely the B.

## 5. Conclusions

The present study showed that the ABI is a robust and valid tool for use with a Finnish-speaking population. It distinguishes well between healthy and dysphonic voices. The threshold value for breathiness in Finnish healthy and dysphonic voices was 2.68.

## Figures and Tables

**Table 1:** Number of dysphonic participants and their diagnoses.

Diagnosis	Number
Functional dysphonia	30
Paralysis/paresis of vocal fold	23
Laryngeal dystonia	23
Other diseases of vocal fold or larynx/other undefined dysphonia	10
Chronic laryngitis	9
Nodules	5
Larynx irritable with voice symptoms	2
Cough with voice symptoms	1
Polyp of vocal cord	1
Laryngeal spasm	1
Dysphagia with voice symptoms	1
Ehlers–Danlos syndrome	1
Larynx trauma	1
Total	108

**Table 2:** Mean values of ABI and perceptual rating of breathiness in non-dysphonic (participants with no diagnosis of dysphonia) and dysphonic groups (participants with diagnosis of dysphonia). Comparison of the groups using Mann–Whitney U test.

	*N*	Mean	SD	Min	Max	*p*-Value
ABI Non-dysphonic Dysphonic						
	87	2.26	1.15	−0.70	4.58	<0.001
	108	3.07	1.75	−0.91	9.38	
Perceptually assessed BNon-dysphonic Dysphonic	87	0.37	0.35	0	1.25	<0.001
	108	1.26	0.74	0	3	

**Table 3:** The results of nine ABI-validated languages showing ABI thresholds, sensitivities, specificities, likelihood ratios, and correlation values r_s_ between the ABI and perceptual evaluation of breathiness (B).

Language	ABI Cut-Off Value	Sensitivity %	Specificity %	LR+	LR−	Correlation r_s_ between ABI and B
Dutch	3.44	82	93	11.63	0.19	0.84
German	3.42	72	90	7.40	0.31	0.86
Japanese	3.44	76	94	8.09	0.13	0.89
Korean	3.69	88	86	6.47	0.14	0.87
Brazilian Portuguese	3.13	88	91	−0.03	0.13	0.87
Spanish	3.40	74	95	16.02	0.27	0.83
South Indian	3.66	62	95	12.19	2.48	0.76
Persian	2.97	70	87	5.44	0.35	0.74
Finnish	2.68	80	80	4.00	0.25	0.82

## Data Availability

The data presented in this study are available on request from the corresponding author. The data are not publicly available due to privacy or ethical restrictions.
